# New Insights Into the Social Rumor Characteristics During the COVID-19 Pandemic in China

**DOI:** 10.3389/fpubh.2022.864955

**Published:** 2022-06-27

**Authors:** Wei Lv, Wennan Zhou, Binli Gao, Yefan Han, Han Fang

**Affiliations:** ^1^School of Safety Science and Emergency Management, Wuhan University of Technology, Wuhan, China; ^2^Department of Hyperbaric Oxygen Treatment Center, Sichuan Academy of Medical Sciences and Sichuan Provincial People's Hospital, University of Electronic Science and Technology of China, Chengdu, China; ^3^School of Architecture, Southwest Jiaotong University, Chengdu, China

**Keywords:** rumor, COVID-19 pandemic, time evolution characteristics, spatial and temporal characteristics, network characteristics

## Abstract

**Background:**

In the early stage of the COVID-19 outbreak in China, several social rumors in the form of false news, conspiracy theories, and magical cures had ever been shared and spread among the general public at an alarming rate, causing public panic and increasing the complexity and difficulty of social management. Therefore, this study aims to reveal the characteristics and the driving factors of the social rumors during the COVID-19 pandemic.

**Methods:**

Based on a sample of 1,537 rumors collected from Sina Weibo's debunking account, this paper first divided the sample into four categories and calculated the risk level of all kinds of rumors. Then, time evolution analysis and correlation analysis were adopted to study the time evolution characteristics and the spatial and temporal correlation characteristics of the rumors, and the four stages of development were also divided according to the number of rumors. Besides, to extract the key driving factors from 15 rumor-driving factors, the social network analysis method was used to investigate the driver-driver 1-mode network characteristics, the generation driver-rumor 2-mode network characteristics, and the spreading driver-rumor 2-mode characteristics.

**Results:**

Research findings showed that the number of rumors related to COVID-19 were gradually decreased as the outbreak was brought under control, which proved the importance of epidemic prevention and control to maintain social stability. Combining the number and risk perception levels of the four types of rumors, it could be concluded that the Creating Panic-type rumors were the most harmful to society. The results of rumor drivers indicated that panic psychology and the lag in releasing government information played an essential role in driving the generation and spread of rumors. The public's low scientific literacy and difficulty in discerning highly confusing rumors encouraged them to participate in spreading rumors.

**Conclusion:**

The study revealed the mechanism of rumors. In addition, studies involving rumors on different emergencies and social platforms are warranted to enrich the findings.

## Introduction

In December 2019, an outbreak of COVID-19, caused by infection with SARS-CoV-2, was found and publicly reported in China and subsequently became a global pandemic (1–5). As of 31 May 2020, more than 5.9 million cases were confirmed in most countries and territories worldwide, with more than 367,000 fatalities (6), seriously threatening the public's lives and health and menacing economic stability and social security of nations.

Public health emergencies are often accompanied by the Internet public opinion crisis characterized by frequent rumors, especially when official authorities' information is delayed or lacking (7). Since the advent of Web 2.0 technology, Internet social media platforms, such as Weibo (similar to Twitter), have gradually replaced traditional media as the dominant platforms for the Chinese public to express their opinions and participate in social affairs. As of the last quarter of 2020, Sina Weibo had about 521 million active users per month, increasing 10 million over the same period last year (8). Due to social media's easy availability and convenience, information spreads more rapidly and widely through these platforms than its traditional counterparts (9, 10). Moreover, the resulting mass of user-provided content promotes vast recruitment of people around shared interests, worldviews, and narratives, thus influencing the evolution of public opinion (11) and further enabling rumors to thrive. In 2013, the World Economic Forum described web-based rumors as ‘digital wildfire' and accentuated the risks they pose to modern society (12). “Drinking ardent spirits can prevent SARS-CoV-2 infection” was a typical example of a rumor spread widely during the outbreak of COVID-19 in China. Since the epidemic outbreak, social media users rushed to the Internet to seek methods of preventing and treating COVID-19. Due to strong concerns about their own lives and the lack of awareness of the disease, many microbloggers distorted the causal relationship between COVID-19 and drinking, and these blogs became very pervasive (13). These false messages were widely discussed on the Internet at the time, causing great chaos and scares.

Compared with the severe acute respiratory syndrome (SARS) outbreak 17 years ago, the COVID-19 outbreak has sparked more rumormongering. Rumors such as ‘Dual yellow oral liquid inhibited novel coronavirus,' ‘The number of confirmed cases of COVID-19 and deaths in a county,' and ‘Some places have been blockaded or the supermarkets have been closed down' sparked panic among the public, triggering people to snap up supplies and posing a severe challenge to the governance of Internet public opinion in the context of the pandemic. Therefore, it is significant to study the characteristics of rumor generation and propagation on the network platform and propose appropriate approaches to suppress rumors effectively.

The following paper is organized as follows: Section Related Work introduces related works of experts in correlative areas and states research gaps. Section Data and Methods provides an introduction to the data collection, data preprocessing, and analysis methodology. In SectionResults and Discussion, the main results are presented and discussed. Section 5 and Section Strengths and Limitations introduce the strengths and limitations of this study, respectively. Finally, a summary of this work and some meaningful conclusions are presented in Section Conclusion.

## Related Work

False information is generally referred to as information pollution. According to the heterogeneity, Meel and Vishwakarma (14) divided false information into several categories: rumor, fake news, misinformation, disinformation, etc. There are different definitions of rumor in pieces of literature. Gist (15) proposed that rumors have three characteristics: word of mouth, informative, and expressive. With the development of information technology, the transmission mode of rumors has changed radically, and they are no longer passed by word of mouth, but they are more prone to distortion of information. The informative and expressive qualities of rumors, however, have not changed. The informative nature of rumors means that they are intended to convey information about particular people, happening, or conditions. Rumors express and satisfy the emotional needs of the group. Based on this, this paper defines rumors as statements that are fabricated and publicized remarks through specific means without a corresponding factual basis.

It is necessary to carry out a series of research on rumors for rapid damage control. At present, there are four important perspectives of rumor analysis: source detection (16–18), propagation dynamics (19, 20), fake information detection (21–23), containment, and intervention (24, 25). A widely spread rumor or fake news can lead to reputation ruin (26), political consequences (27), and economic loss (28). Numerous scholars are dedicated to revealing the inherent driving mechanisms of rumors and putting forward effective strategies to prevent and control rumors (29–32). The in-depth tracing of misinformation and disinformation across social networks is a complicated process. Bruns et al. (33) drew on a mixture of quantitative and qualitative methods and showed the dynamics of the rumor to uncover the main driving forces and inflection points of COVID/5G conspiracy theories. Islam et al. (34) followed and examined COVID-19–related rumors on various online platforms and further explored their impacts on public health.

When an emergency occurs, many factors drive the generation and transmission of rumors, such as psychology (35), the heterogeneity of individual behaviors (36), opinion leaders (37), etc. There is a complex network relationship between rumors and driving factors. Wang et al. (38) discovered that network topology is an important factor affecting the spread of rumors. Dong et al. (39) found that the angrier the public feel, the more rumors there will likely be. Hui et al. (40) drew an interesting conclusion that the education level of the crowd is an essential factor affecting the final scale of rumor propagation.

There are subtle differences between the rumor generation and dissemination mechanisms. Most studies on rumor influencing factors have been carried out in the propagation mechanism, and plenty of models and methods have been implemented to simulate the spreading process of rumors, such as epidemic models (41–43), forest fire model (44), and explosive explosion principle (45). Some scholars also use questionnaire methods to study the influencing factors of rumors. Sun et al. (46) investigated 556 Chinese people through online questionnaires and obtained the factors influencing middle-aged and older adults regarding the re-spreading of rumors about COVID-19. Experiments showed that network properties profoundly affect the rumor diffusion process, and the complex structure of social networks can be modeled using different graphical formats. Cheng et al. (47) utilized the stochastic epidemic model to analyze rumor propagation on the online social site BlogCatalog by formalizing an undirected graph G (V, E) dataset, which contains 10,312 nodes and 333,983 edges. Wood (48) analyzed the content and social network of 25,162 original tweets about the Zika virus conspiracy theory, pointing out that conspiracy theories spread through a more decentralized network relative to debunking messages. However, the behaviors of rumor makers are the root of curbing rumors and are an effective way to block rumors. Few studies have investigated the mechanism of rumor generation systematically. Zhu and Liu (49) put forward the disinformation behavior evolution model of the rumor maker by the system dynamics, and concluded that the effect of personal effects on rumor tendency is the most significant, followed by social effects, while the government effects have only a subtle effect on the rumor tendency. In summary, the mechanics of online rumors is very complicated and dynamic, making it difficult to prevent a rumor from generating and propagating throughout the Internet. A great deal of previous research on rumors has focused on the spread of rumors. There has been no detailed investigation regarding the rumor generation mechanism.

On the basis of extant literature, it can be argued that the category of false information has been divided (14, 50). Many different factors are available for classifying rumors, such as type, scope, and characteristics. Jaeger et al. (51) classified rumors into believable and unbelievable based on their credibility. Zubiaga et al. (22) had interestingly split rumors into two main categories: long-standing rumors and breaking news rumors. From the perspective of psychology, Knapp (52) categorized rumors into “pipe-dream” rumors, “bogy” rumors, and “wedge-driving” rumors. Therefore, the classification of rumors is not yet uniform. Only a handful of studies have classified rumors during COVID-19 (53). This leads to the first research question (RQ),

**RQ1.:** *What are the categories of online rumors related to the epidemic, and how to classify them? What are the characteristics of different types of rumors?*

As the country was first hit by the pandemic, China experienced a complex social environment during the severe anti-pandemic period. Exploring the influencing factors and trends of related rumors has positive significance for the managers to maintain social stability. Zhu et al. (54) analyzed social media topics and emotional change characteristics during COVID-19 from spatiotemporal perspectives. Chen et al. (53) divided the COVID-19 outbreak into five periods according to the key events and disease epidemic and plotted the trends of the epidemic and the focus of the public at different stages. Motivated by these studies, we incorporate the trend of epidemic and essential news into rumor studies. Thus, the follow-up research question is,

**RQ2.:** *What is the trend of rumors during the outbreak? What factors affect rumors?*

However, emergencies are not homogenous. For instance, the factors and variables that have influenced the rumors related to the Middle East respiratory syndrome outbreak in 2015 and the Zika virus in 2015-16 are different (48, 55). Besides, a lack of research specifies the differences between the causal factors of different types of rumors. More importantly, limited studies have attempted to distinguish between rumor making and rumor spreading. From this, we get the third research question,

**RQ3.:** *What are the drivers of rumors? What are the similarities and differences between rumors' generating and spreading factors? What are the characteristics of the causal factors of different types of rumors?*

To answer this, this study identified 1,537 rumor-related posts about COVID-19 on Weibo, one of China's most popular social media platforms. We explored the classification and characteristics of rumors. The development trend of emergencies and hot events were also taken into account to investigate the spatiotemporal characteristics of rumors. Besides, rumor-driving factors were extracted and discussed.

## Data and Methods

This section presents the data and methods, including data collection, data processing, and the three main research methods. In the data processing section, we explore RQ1.

### Data Collection

*Rumor data cases:* Sina Weibo set up an official account for dispelling false information on 18 November 2010. During the outbreak, this account continuously released and forwarded relevant refutation information. Therefore, we collected 1,537 rumor data cases related to the COVID-19 pandemic released by this account from 1 January to 31 May 2020 (a total of 152 days). The interval of data source covered the day after the WHO officially announced the presence of the novel virus to the day when China had basically controlled the domestic epidemic. The data case involved the rumored statement, the release time, and the region information.

*The cumulative confirmed cases:* The number of cumulative confirmed cases in each city of China on 31 May 2020 was recorded from TouTiao's column of the real-time epidemic of new coronavirus. Since 11 January 2020, China's National Health Commission began to report on pneumonia of unknown cause, so the cumulative daily number of confirmed cases nationwide from 10 January to 31 May 2020 were collected from the China National Health Commission website.

*GDP index in 2019:* Based on the 2019 Provincial National Economic and Social Development Statistical Communiqués released in 2020, each city's GDP index in 2019 was also collected.

### Data Preprocessing

The relevant information of 1,537 rumors was collected extensively, such as the motives of rumormongers, the content of the text, social consequences, etc. It was found that some social haters were trying to tarnish the government's image through rumors. At the same time, the unknown pneumonia virus caused a great deal of social panic. The lack of information led to speculation about confirmed cases and the status of the epidemic. People were prone to believe in various prescriptions to combat the virus. The real news from one place was spread around the country through alterations with ulterior motives. As a result, the 1,537 rumors were first divided into four categories, Creating Chaotic-type, Creating Panic-type, Pseudoscientific-type, and Other False-type, which could be coded as CC, CP, P, and O, respectively. The feature of each type is described in Table 1.

**Table 1:** Four categories of the collected rumors.

**The rumor type**	**Code**	**Description and explanation**
Creating Chaotic	CC	Mainly manifested as slandering the government's image, instigating the relationship between the government and society, endangering the law's implementation, or undermining social stability.
Creating Panic	CP	Mainly manifested in spreading false epidemic information and causing social panic, which can be divided into (a) forging local suspected confirmed cases, (b) exaggerating the tragic situation of the epidemic, (c) importing cases from Wuhan, (d) importing cases from abroad.
Pseudoscientific	P	Mainly manifested in the promotion of various false epidemic prevention or anti-epidemic methods.
Other False	O	Mainly manifested as quoting the actual situation or news from one area to another, which is very confusing.

To investigate the public's cognition regarding the harmful degree of different rumors, a survey in China was conducted in April 2020. The survey questionnaire (see Appendix) was distributed via the Tencent Questionnaire platform. Respondents could fill in, submit, and share the questionnaire by a QR code or forwarding link generated by the platform. The survey finally collected a sample of 145 participants (96.02% effective rate) (56), which varied in demographic characteristics, such as gender (39% men, 61% women), age (10% under 18 years, 54% 18–30 years old, 15% 31–40 years old, 14% 41–50 years old, 5% 41–50 years old, and 2% over 60 years old), occupation (37% civil servants, 7% enterprise employees, 2% self-employed, 42% students, and 12% other), and education (19% junior high school and below, 10% high school and college, 47% undergraduate, and 24% postgraduate and above). In this survey, participants first answered questions assessing their personality traits. Then, they read 12 false rumors and rated the harm of each one. The scale was a five-point Likert scale (57), ranging from 1 (‘no harm') to 5 (‘highly harmful'). According to the characteristics and manifestations of each type of rumor, 12 rumors included 4 CC-type rumors, 4 CP-type rumors, 2 P-type rumors, and 2 O-type rumors separately. The value of Cronbach's alpha was 0.909, and the KMO value was 0.900, which indicated that the questionnaire passed the reliability and validity test and could be further analyzed. Then the average risk perception score of all kinds of rumors could be calculated based on the questionnaire data. The description, score, and average value of each rumor are presented in Table 2.

**Table 2a:** Risk scoring and mean for each rumor.

**Rumor's type**	**The number of each score**					**Mean**
	**1**	**2**	**3**	**4**	**5**	
**CC-type rumors**						**3.667**
Yu Tian, the South China Seafood Market owner, whose father-in-law is vice-chairman of the Wuhan Huanan Seafood Market.	19	21	41	29	35	3.276
The medical team of Shanghai aid Hubei has no food to eat, only instant noodles.	12	14	36	35	48	3.641
Huang Yanling, a graduate of Wuhan Institute of Virology, Chinese Academy of Sciences, is the first “Patient Zero” infected with the new coronavirus.	15	18	22	34	56	3.676
Many other places are underreporting confirmed cases, possibly amounting to tens of thousands.	5	12	21	36	71	4.076
**CP-type rumors**						**3.864**
Zhaizhuang village, Xushui District, Baoding City has three cases of infection, and the village has been closed.	8	15	45	33	44	3.621
There are untreated corpses in a hospital in Wuhan.	7	8	15	31	84	4.221
The man is infected with COVID-19 after a 10-min stop in Wuhan.	6	13	25	37	64	3.966
There are six people coming back from Korea in the qianshuiwan community of Tieling City.	7	20	36	36	46	3.648
**P-type rumors**						**3.700**
Zhong Nanshan released that drinking more Dancong tea has a great effect on preventing pneumonia.	15	20	29	39	42	3.503
The N95 mask, which has been used for seven days, can continue to be used after being blown with a hairdryer or disinfected with alcohol.	8	13	26	37	61	3.897
**O-type rumors**						**3.555**
Everyone needs to stay at home from 4:00 to 4:30 this afternoon. There will be planes spraying disinfectant.	13	15	37	40	40	3.545
A house was ignited by improper use of disinfectant alcohol in a community in Chengdu.	11	15	43	33	43	3.566

*The bold values represent the average risk scores of each type of rumors*.

**Figure 1:**
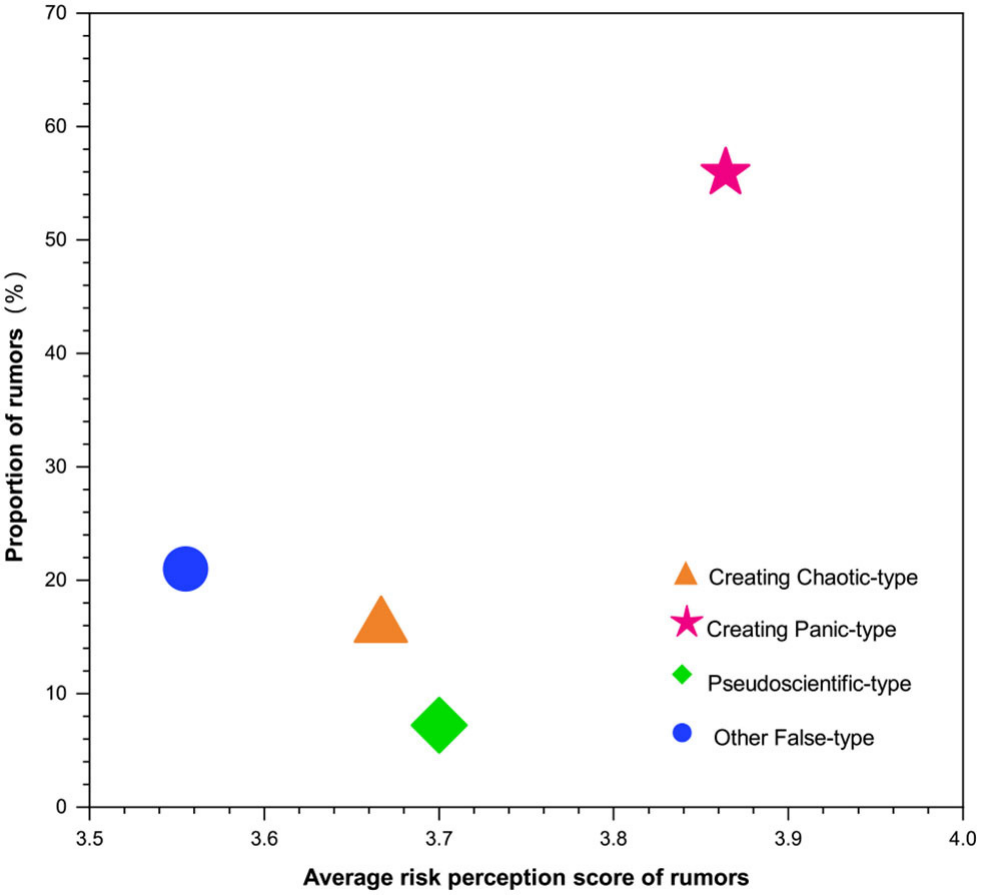
Risk patterns of four types of rumors.

Figure 1 shows the risk pattern of the four types of rumors. The x-axis represents each type of rumor's average risk perception score calculated according to the questionnaire data. The y-axis represents the proportion of each type of rumor among the 1,537 rumors collected. From the figure below, the risk index of CP-type rumors was the highest, which is why the government and social organizations prioritized dispelling such rumors.

### Methods

#### Time Evolution Analysis

In order to explore the variation feature of the four types of rumors in the time dimension, the number of rumors of each type was counted by day. Then the number curves were drawn to show the peak point, duration, and extinction time of each type of rumor. Meanwhile, the similarities and differences among the four types of rumors were also compared and analyzed. The time evolution analysis could help us understand the vitality of various rumors and distinguish the characteristics of different rumors.

#### Correlation Analysis

Research on the related factors of rumors may play a positive role in the intervention of rumors. Based on the collected data of the cumulative number of confirmed cases and each city's GDP index, it was appropriate to analyze the correlation between rumors and various factors from the spatial and temporal distribution of rumors (57). This study mainly explored the influence of economic development and the region's pandemic situation on the rumor and analyzed the rumor's spatial distribution during the pandemic period in China. Considering that the temporal scale could be represented by the cumulative number of the confirmed cases during the period, the changing trend of the number of rumors could be divided into several phases. Then the effects of the COVID-19 pandemic status and the related events reported on the changing number of rumors would be analyzed separately. The correlation analysis was conducted by SPSS 26.0, and the Pearson correlation coefficient was used to test the statistical correlation (58–60). A *p*-value <0.05 was set for statistical significance (61–63).

#### Network Analysis

As is known to all, there are many complex factors behind rumors. In fact, a rumor can be driven by one or more reasons, and at the same time, one reason can induce a variety of rumors. There may be a complex connection between the variety of rumors and driving factors. Through network analysis technology, it is possible to construct a network of the driving factors and the rumors and reveal the importance or the key effect of the various factors in the network. Therefore, an analysis of the driver of each rumor case was the first work to be conducted. Then these reasons were divided into different categories according to the attribute similarity. Next, taking reason and rumor as nodes, and taking the common reason or common rumor as a relationship, 1-mode network of the rumors,1-mode network of the reasons, and 2-mode network of the rumors-reasons could be built and analyzed in depth.

## Results and Discussion

In this section, the time evolution characteristics, the spatial and temporal correlation characteristics, and the network characteristics of the rumors are analyzed and mined (**RQ2** and **RQ3**).

### Time Evolution of the Rumors

**Figure 2:**
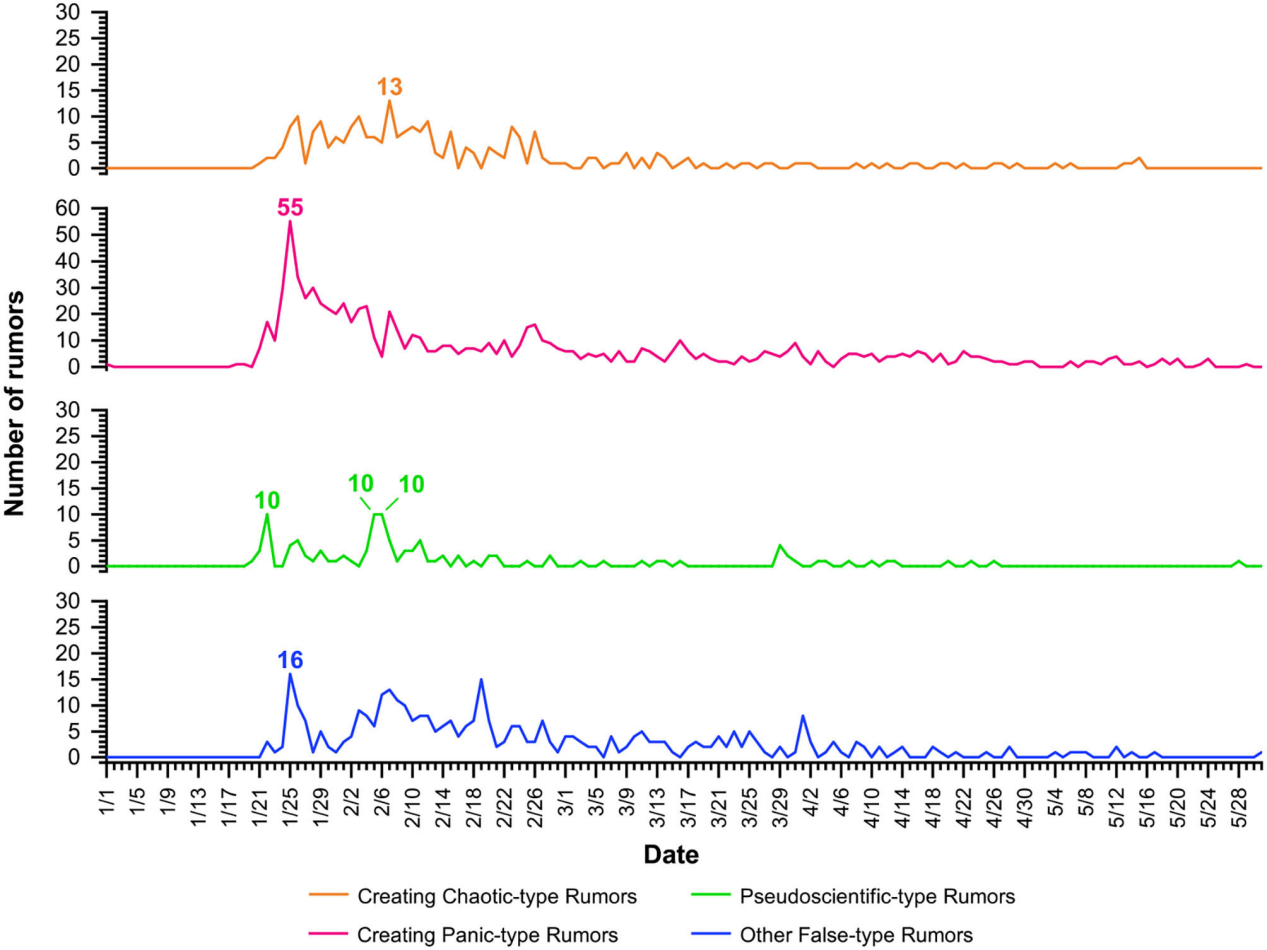
The variation in the four types of rumors over time.

Figure 2 shows the trend graphs of the four types of rumors. From the graph below, the CC-type rumors were relatively frequent between the end of January and the end of February, with the largest number reaching 13 a day. In late January, the number of CP-type rumors began to rise sharply, and in an interview with the official media, Zhong Nanshan, an academician of the Chinese Academy of Engineering, confirmed for the first time that the virus could be “human-to-human” and revealed that 14 health care workers had been infected. Then, the whole of Wuhan city was blocked. A series of factors, such as mass migration during the Spring Festival and the public awareness of the new virus, caused great panic and a psychological influence on the public, which contributed to the number of such rumors sharply increasing in the following 11 days. At its peak, 55 such rumors appeared in 1 day. The P-type rumors mainly appeared in the early stages of the COVID-19 pandemic, but due to the timely popularization of the knowledge of the new coronavirus, the number of such rumors had also declined. The O-type rumors scattered at all stages of the epidemic. Such rumors were mainly quoted from some unconfirmed information such as ‘Alcohol disinfection triggered Fire,' or some rumors debunked but still spreading.

### Spatial Correlation of the Rumors

According to the text description and release area of 1,537 rumors, we counted the number of times each city was affected by rumors, and sorted out the cumulative number of confirmed cases and the 2019 GDP index of these cities (unit: billion RMB), as presented in Table 2. The result illustrated that Wuhan was not only the hardest-hit area of the pandemic but also the birthplace and agglomeration area of rumors. At the same time, the development level of Wuhan city was also at the forefront of the country.

**Table 2b:** The number of rumors, cumulative confirmed cases, and 2019 GDP index of each prefecture-level city (part).

**Name of the city**	**Cumulative number of rumors**	**2019 GDP index(billion)**	**Cumulative number of confirmed cases**
Wuhan	300	16223.21	50340
Shanghai	56	38155.32	672
Beijing	50	35371.30	593
Chongqing	33	23605.77	579
Wenzhou	41	6606.11	504
…	…	…	…
Jiaozuo	1	2761.10	32
Puyang	1	1581.49	17
Binzhou	1	2457.19	15
Dezhou	1	3022.27	37
Puer	1	875.28	4

From the correlation analysis results in Table 3, it can be seen that there was a significant positive correlation between the cumulative number of rumors and the cumulative number of confirmed cases from the perspective of spatial distribution. A weak positive correlation existed between the cumulative number of rumors and the GDP index, as well as between the cumulative number of confirmed cases and the GDP index. These results indicated that the cumulative number of confirmed infection cases might be the most critical factor in inducing rumors. In other words, the more confirmed infection cases in an area, the more rumors might emerge in that area.

**Table 3:** Correlation analysis result from the perspective of spatial dimension.

		**Cumulative number of rumors**	**GDP index (in 2019)**	**Cumulative number of confirmed cases**
Cumulative number of rumors	Pearson correlation	1	0.359**	0.874**
	Sig. (2-tailed)		0.000	0.000
	N	295	295	295
GDP index (in 2019)	Pearson correlation	0.359**	1	0.181**
	Sig. (2-tailed)	0.000		0.002
	N	295	295	295
Cumulative number of confirmed cases	Pearson correlation	0.874**	0.181**	1
	Sig. (2-tailed)	0.000	0.002	
	N	295	295	295

** *Significant at the 1% level. The bold values represent the Pearson correlation coefficient values*.

### Temporal Correlation of the Rumors

Since the official website of the National Health Commission of the People's Republic of China began to report the cases of new coronavirus pneumonia daily from 11 January 2020 onward, the correlation analysis was conducted using the data between 10 January 2020 and 31 May 2020 (143 days in total). The variables analyzed included the number of confirmed cases per day, the number of new confirmed cases, the number of accumulated rumors, and the number of new rumors per day. Different from the spatial correlation analysis, using the data day by day nationwide, the correlation between the variables was investigated from the perspective of the temporal dimension. Table 4 summarizes the results of the temporal correlation analysis.

**Table 4:** Correlation analysis results from the perspective of the temporal dimension.

**Variable**	**Average Value**	**N**	**Cumulative confirmed cases**	**New daily confirmed cases**	**Cumulative rumors**	**New daily rumors**
Cumulative confirmed cases	64,764.71	143	1			
New daily confirmed cases	584.29	143	−0.258**	1		
Cumulative rumors	1,076.19	143	0.954**	−0.292**	1	
New daily rumors	10.78	143	−0.483**	0.478**	−0.493**	1

** *Significant at the 1% level. The bold values represent the Pearson correlation coefficient values*.

**Figure 3:**
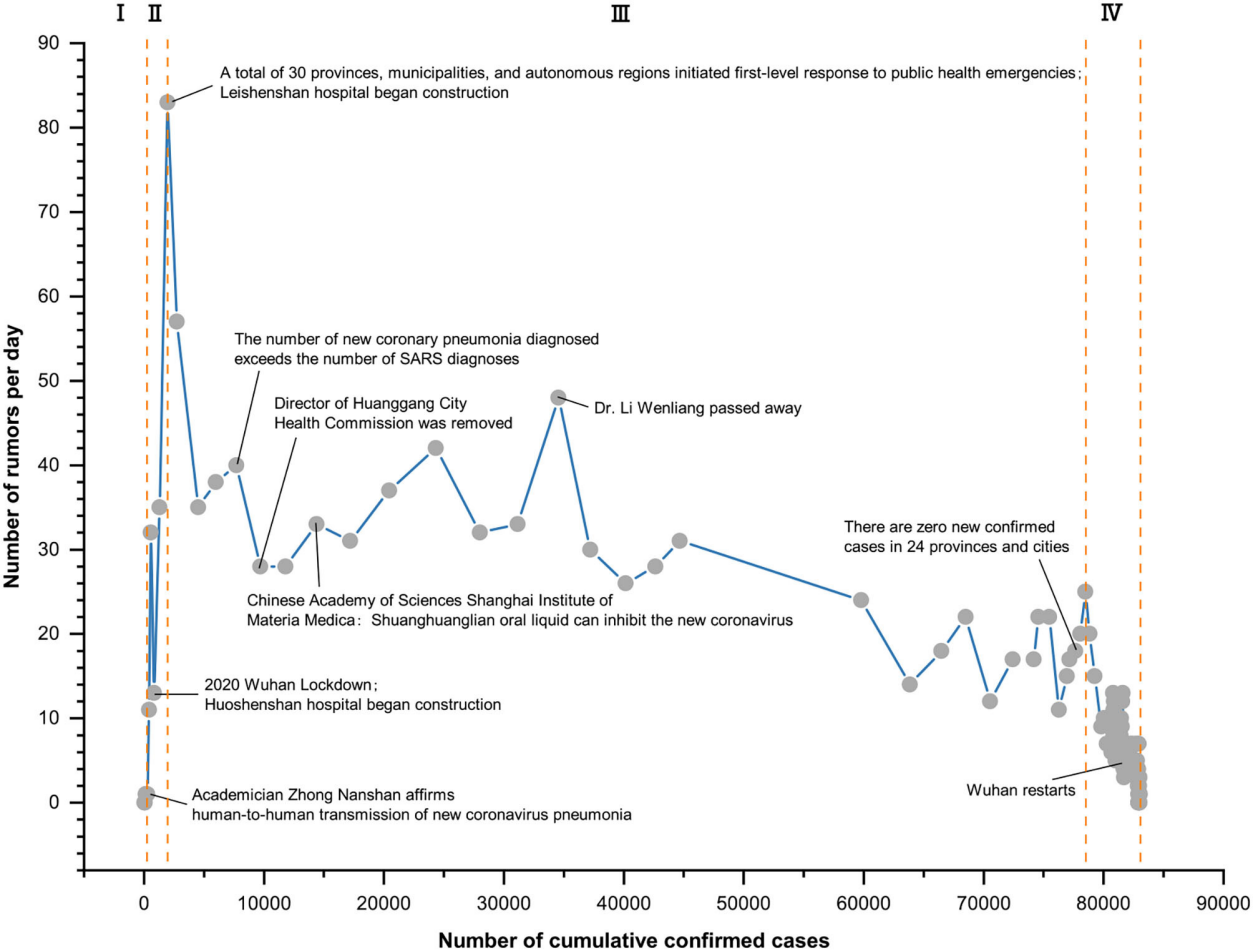
Point figure chart of the daily rumor number and the cumulative confirmed cases.

From Table 4, to be exact, the number of the new daily rumors had a significant negative correlation with the cumulative number of confirmed infection cases nationwide (*r* = −0.483^**^) and the cumulative number of rumors (*r* = −0.493^**^), while a positive correlation with the number of the new daily confirmed cases (*r* = 0.478^**^). The cumulative number of rumors highly positively correlated with the cumulative confirmed cases (*r* = 0.954^**^). These results confirmed that the confirmed cases, indeed, positively affected the number of rumors not only by cumulative but also by day. Meanwhile, the negative correlations reflected a downward trend of the new daily rumors in the time dimension, which also can be shown in Figure 3.

**Figure 4:**
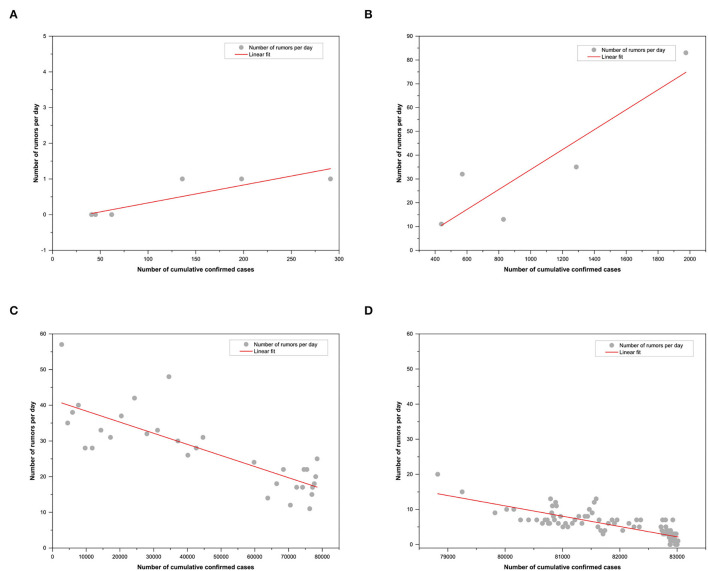
The linear fitting for the four stages. **(A)** The first stage; **(B)** The second stage; **(C)** The third stage and **(D)** The fourth stage.

According to the trend of the daily number of rumors over time in Figure 3, it can be divided into four stages: the first stage of the incubation period from 10 January to 20 January, the second stage of the outbreak period from 21 January to 25 January, the third stage of the development period from 26 January to 26 February, and the fourth stage of the retreat period from 27 February to 31 May. Figure 4 and Table 5 present the staged fitting results of the four stages, respectively. Combining the development and changes of the pandemic situation and the actual related events in each stage, the change in rumor quantity was analyzed as follows.

**Table 5:** Linear fitting parameters of the four stages.

	**The first stage**	**The second stage**	**The third stage**	**The fourth stage**
N	11	5	32	95
Df	9	3	30	93
Sum squared residual	0.38364	635.90865	1221.52612	454.72589
Pearson's r	0.90784	0.90081	−0.81009	−0.81111
R-square(COD)	0.82417	0.81146	0.65624	0.6579
Adj. R-square	0.80463	0.74861	0.64479	0.65422
F-value	42.18464	12.91172	57.27121	178.84641
Sig.	1.12105E-4	0.03694	1.94145E-8	0

The incubation period of rumors is from 10 January to 20 January 2020. As shown in Table 5, there was a significant positive correlation between the number of rumors and the cumulative confirmed cases (*r* = 0.908^**^). During this period, the daily number of rumors was only one or two, which was related to the fact that on 11 January, the Wuhan Health Commission announced that there was no evidence of infection among medical staff and human-to-human transmission. From 12 January to 16 January, the Wuhan Health Commission continuously reported no new confirmed cases, which indicated that although there was news of pneumonia of unknown etiology at this stage, the overall social attention was low. The quantity of rumors related to it was relatively small.

The outbreak period of rumors was from 21 January to 25 January 2020. As shown in Table 5, there was a significant positive correlation between the daily number of rumors and the cumulative confirmed cases (*r* = 0.901^**^). At this stage, the daily number of rumors increased sharply. On the evening of 20 January, Academician Zhong Nanshan accepted an interview with reporters, and then “Zhong Nanshan affirms the human-to-human transmission of new coronavirus pneumonia” became a trending topic on Sina Weibo, a turning point in the public opinion of the novel coronavirus. After this turning point, the public's concern about it rose sharply, and the rumors had also surged. Subsequently, Wuhan went into lockdown on 23 January, and a total of 30 provinces and autonomous regions nationwide activated first-level public health emergency response on 25 January. The daily number of rumors also peaked at 83 on 25 January 2020.

The development period of rumors was from 26 January to 26 February 2020. As shown in Table 5, there was a significant negative correlation between the number of rumors and the cumulative confirmed cases. Figure 4C shows that the number of rumors decreased volatility with the cumulative growth of confirmed cases. There had been a shift from a positive correlation to a negative correlation, indicating that the government's series of measures had gradually emerged. This trend was affected by many social events, such as ‘Doctor clove and other Internet platforms have set up rumor refuting columns,' ‘the number of newly diagnosed cases in 24 provinces and cities was zero on February 24,' and ‘the number of newly cured cases in China exceeded the daily number for the first time on February 18.'. However, under the overall good situation, the number of rumors also fluctuated, which was related to social hot events, such as ‘Red Cross Society of China Hubei Branch announced that the use of donated materials raises questions,' “Citizens in many places snap up Shuang Huang Lian oral liquid,” and “Doctor Li Wenliang's death on February 7.”

The fading period of rumors was from 27 February to 31 May 2020. As shown in Table 5, there was a significant negative correlation between the number of daily rumors and the cumulative confirmed cases. Figure 4D shows that the number of daily rumors was below 20 and stabilized below 10 after 2 April 2020. As Wuhan got lockdown relief at midnight on 8 April in response to the slowing of the outbreak, ‘Resume study' and ‘Resume work' became hot topics of public discussion. The government's strict control over imported cases had also effectively prevented China's international pandemic crisis.

### Social Network Analysis of the Rumors

#### Analysis of Rumor Drivers

By means of analyzing the motivations and referring to relevant literature, three categories with 15 rumor-driving factors were extracted from the collected 1,537 rumors data. The rumor causation categories included generative factors (the factors that can generate rumors), spreading factors (the factors that can spread rumors), and generative-spreading factors (the factors that can both generate and spread rumors). The 15 driving factors were divided and coded according to the different classification characteristics, as listed in Table 6.

**Table 6:** Driving factors of the collected rumors.

**Categories**	**Code**	**Driving factors**	**References**
Generative factors	G1	Vent feelings of dissatisfaction	–
	G2	Grab economic interests	(14, 43)
	G3	Self-hype, harvesting Fans	(14)
	G4	To fool the public and seek the social presence	–
	G5	Deliberate sabotage by hostile forces	(14)
	G6	Slander others maliciously	–
Spreading factors	S1	The confusing nature of rumors	(64)
	S2	Low scientific literacy of the public, challenging to distinguish rumors	(46, 65, 66)
	S3	Promotion of public figures	(37, 46)
Generative -	B1	Uncertainty in the development of the epidemic	(46)
Spreading	B2	Panic psychology	(46, 53, 67, 68)
factors	B3	Imperfect laws and regulations, inadequate network supervision	(69)
	B4	Relatively lagging in information disclosure	(65, 68)
	B5	The government mishandled and broke the people's trust	(65, 68, 69)
	B6	Well-intentioned reminder, arouse the public's attention to the epidemic	–

Among the 1,537 rumor data collected, only the relevant government information circulars of 757 rumors were found, and the drivers for the rumor were extracted. These rumors were numbered according to their classifications: CC1–CC162 represent the Creating Chaotic-type rumors, CP1–CP368 represent the Creating Panic-type rumors, P1–P66 represent the Pseudoscientific-type rumors, and O1–O161 represent the Other False-type rumors. Table 7 shows the part relationship between rumors and driving factors.

**Table 7:** Relationship between rumor and driving factors (part).

**Rumor code**	**Rumor driving factors**	
	**Generative factors**	**Spreading factors**
CC1	G1_‵_ G4_‵_ G5_‵_ G6_‵_ B2_‵_ B3_‵_ B5	S2_‵_ S3_‵_ B1_‵_ B2_‵_ B3_‵_ B4_‵_ B5
CC2	G5_‵_ B1_‵_ B2_‵_ B3_‵_ B4	S2_‵_ B2_‵_ B4_‵_ B5
CC3	G1_‵_ G5_‵_ G6_‵_ B1_‵_ B2_‵_ B4_‵_ B5	S2_‵_ B1_‵_ B2_‵_ B5
CP1	B4_‵_ B5	S2_‵_ B1_‵_ B2_‵_ B4_‵_ B5
CP2	B1_‵_ B2_‵_ B3_‵_ B4	S1_‵_ S2_‵_ B1_‵_ B2_‵_ B4
CP3	B1_‵_ B2_‵_ B3_‵_ B4	S2_‵_ B1_‵_ B2_‵_ B4
——	——	——
P1	G2_‵_ G4_‵_ B2_‵_ B3_‵_ B4	S1_‵_ S2_‵_ B2_‵_ B3_‵_ B4
P2	G2_‵_ G3	S1_‵_ S2
P3	G4_‵_ B1_‵_ B3_‵_ B4	S1_‵_ S2_‵_ B1_‵_ B3_‵_ B4
O1	G3_‵_ B1_‵_ B3_‵_ B4_‵_ B6	S1_‵_ S2_‵_ S3_‵_ B3_‵_ B6
O2	G3_‵_ B3	S1_‵_ S3_‵_ B3
O3	B1_‵_ B2_‵_ B4_‵_ B6	S2_‵_ B1_‵_ B2_‵_ B6

#### Network Construction

In order to construct the network of the rumors, social network analysis technology was adopted. Table 8 is the affiliation matrix of rumor drivers. In the matrix, *a*_ij_ = 0 means that the *j-*th driving factor will not affect the *i-*th rumor, while *a*_ij_ = 1 means the *j-*th risk factor will lead to the *i-*th risk. In social networks, the degree of centrality of a node is the number of events to which the node belongs. The larger centrality of the node, the more events they belong to, and the more critical the network's position. According to the affiliation relationship matrix of the rumor-rumor drivers, NetDraw software was adopted to visually analyze the driver-driver 1-mode and driver-rumor 2-mode network graphs. Ucinet software was adopted to calculate each driving factor's degree centrality to expose the critical factors leading to the fabricating and spreading of rumors.

**Table 8:** The affiliation matrix of rumor driver (part).

	**G1**	**G2**	**G3**	**G4**	**G5**	**G6**	**B1**	**B2**	**B3**	**B4**	**B5**	**B6**
CC1	1	0	0	1	1	1	0	1	1	0	1	0
CC2	0	0	0	0	1	0	1	1	1	1	0	0
CC3	1	0	0	0	1	1	1	1	0	1	1	0
CP1	0	0	0	0	0	0	0	0	0	1	1	0
CP2	0	0	0	0	0	0	1	1	1	1	0	0
CP3	0	0	0	0	0	0	1	1	1	1	0	0
——	——	——	——	——	——	——	——	——	——	——	——	——
P1	0	1	0	1	0	0	0	1	1	1	0	0
P2	0	1	1	0	0	0	0	0	0	0	0	0
P3	0	0	0	1	0	0	1	0	1	1	0	0
O1	0	0	1	0	0	0	1	0	1	1	0	1
O2	0	0	1	0	0	0	0	0	1	0	0	0
O3	0	0	0	0	0	0	1	1	0	1	0	1

Using Ucinet software, the driver-rumor matrix was converted to the drive-driver matrix, as shown in Table 9 and Table 10. The diagonal value represents the number of rumors connected by a single causal factor. For example, in Table 9, *a*_11_ = 113 means that the driving factor G1 has triggered 113 rumors, and *a*_12_ = 5 means that the driving factors G1 and G2 together lead to five rumors. The same explanation applies to Table 10.

**Table 9:** The 1-mode matrix of the generative factors.

	**G1**	**G2**	**G3**	**G4**	**G5**	**G6**	**B1**	**B2**	**B3**	**B4**	**B5**	**B6**
G1	113	5	9	30	61	22	42	42	34	36	60	7
G2	5	113	30	23	19	8	19	21	54	32	14	6
G3	9	30	107	41	16	6	33	22	38	25	15	9
G4	30	23	41	188	39	8	49	43	45	48	44	10
G5	61	19	16	39	137	24	36	45	40	35	69	6
G6	22	8	6	8	24	38	10	9	19	5	10	1
B1	42	19	33	49	36	10	272	134	75	156	44	52
B2	42	21	22	43	45	9	134	302	69	147	51	74
B3	34	54	38	45	40	19	75	69	230	82	44	31
B4	36	32	25	48	35	5	156	147	82	303	43	67
B5	60	14	15	44	69	10	44	51	44	43	130	11
B6	7	6	9	10	6	1	52	74	31	67	11	143

**Table 10:** The 1-mode matrix of the spreading factors.

	**S1**	**S2**	**S3**	**B1**	**B2**	**B3**	**B4**	**B5**	**B6**
S1	336	233	45	91	143	127	109	53	79
S2	233	545	45	179	336	127	211	100	135
S3	45	45	65	14	20	30	14	22	8
B1	91	179	14	221	169	48	115	44	50
B2	143	336	20	169	432	84	183	80	99
B3	127	127	30	48	84	208	60	43	33
B4	109	211	14	115	183	60	252	49	63
B5	53	100	22	44	80	43	49	142	9
B6	79	135	8	50	99	33	63	9	159

#### Driver-Driver 1-Mode Network Analysis

**Figure 5:**
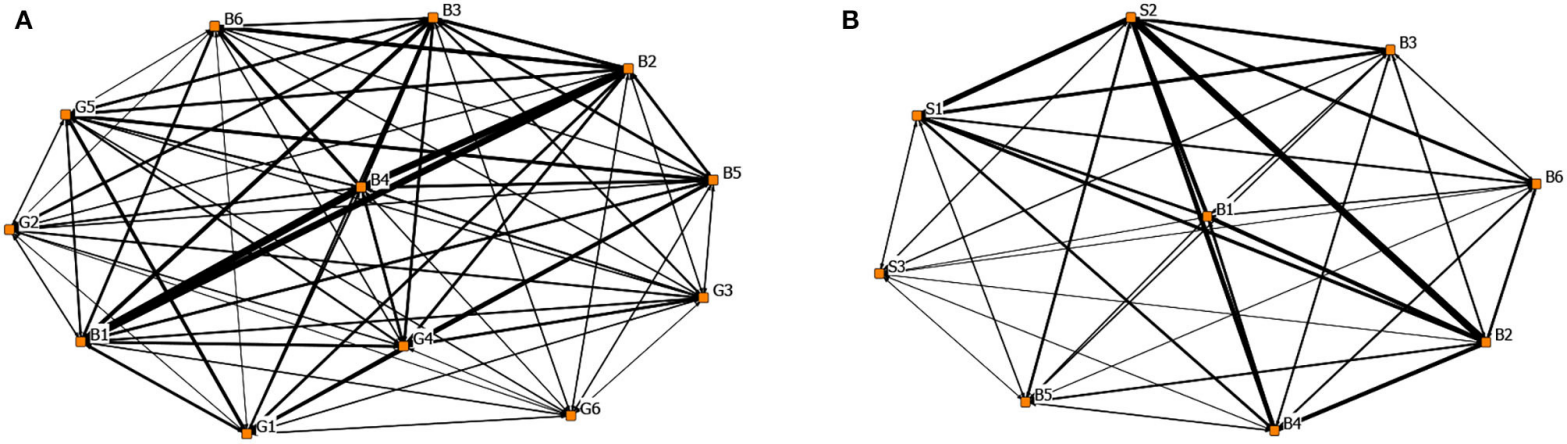
The rumor driver-driver 1-model network graph. **(A)** 1-mode network of the generative factors and **(B)** 1-mode network of the spreading factors.

Figure 5 shows the 1-mode driver-driver network graph of rumor generation factors and propagation factors. In the figure, the edge thickness presents the number of connections between nodes. It can be seen that the generation and dissemination of rumors are the results of the interaction between subjective and objective reasons. Table 11 shows the degree centrality of the 1-mode network. In the process of rumor generation, B4 (Relatively lagging in information disclosure), B2 (Panic psychology), and B1 (Uncertainty in the development of the epidemic) were the main reasons, as shown in Figure 5A and Table 11. A series of related rumors were derived when the novel coronavirus caused significant social changes and due to the lack of knowledge of COVID-19, and the lag of official information release.

**Table 11:** Degree centrality of generative factors and spreading factors.

	**Driving factors code**	**Degree**	**NrmDegree**	**Share**
Generative factors	B4	676.000	39.394	0.138
	B2	657.000	38.287	0.134
	B1	650.000	37.879	0.132
	B3	531.000	30.944	0.108
	B5	405.000	23.601	0.083
	G5	390.000	22.727	0.079
	G4	380.000	22.145	0.077
	G1	348.000	20.280	0.071
	B6	274.000	15.967	0.056
	G3	244.000	14.219	0.050
	G2	231.000	13.462	0.047
	G6	122.000	7.110	0.025
Spreading factors	S2	1366.000	50.818	0.210
	B2	1114.000	41.443	0.171
	S1	880.000	32.738	0.135
	B4	804.000	29.911	0.124
	B1	710.000	26.414	0.109
	B3	552.000	20.536	0.085
	B6	476.000	17.708	0.073
	B5	400.000	14.881	0.062
	S3	198.000	7.366	0.030

Figure 5B and Table 11 illustrate that S2 (Low scientific literacy of the public, challenged to distinguish rumors), B2 (Panic psychology), and S1 (The confusing nature of rumors) were the main factors that promote the dissemination of rumors. They also jointly connected the largest number of rumors. It can be seen that while the public was challenged to distinguish rumors, the deceptive nature of rumors also promoted the spread of rumors.

#### Generating Driver-Rumor 2-Mode Network Analysis

Based on the affiliation matrix of rumor drivers, the driver-rumor 2-mode network graph was visualized by the NetDraw software. In the graphs, the orange square nodes represent the driving factors of the rumors, the green circular nodes represent the rumors, and the node size represents the rumor quantity or the degree centrality of the rumor's drivers.

**Figure 6:**
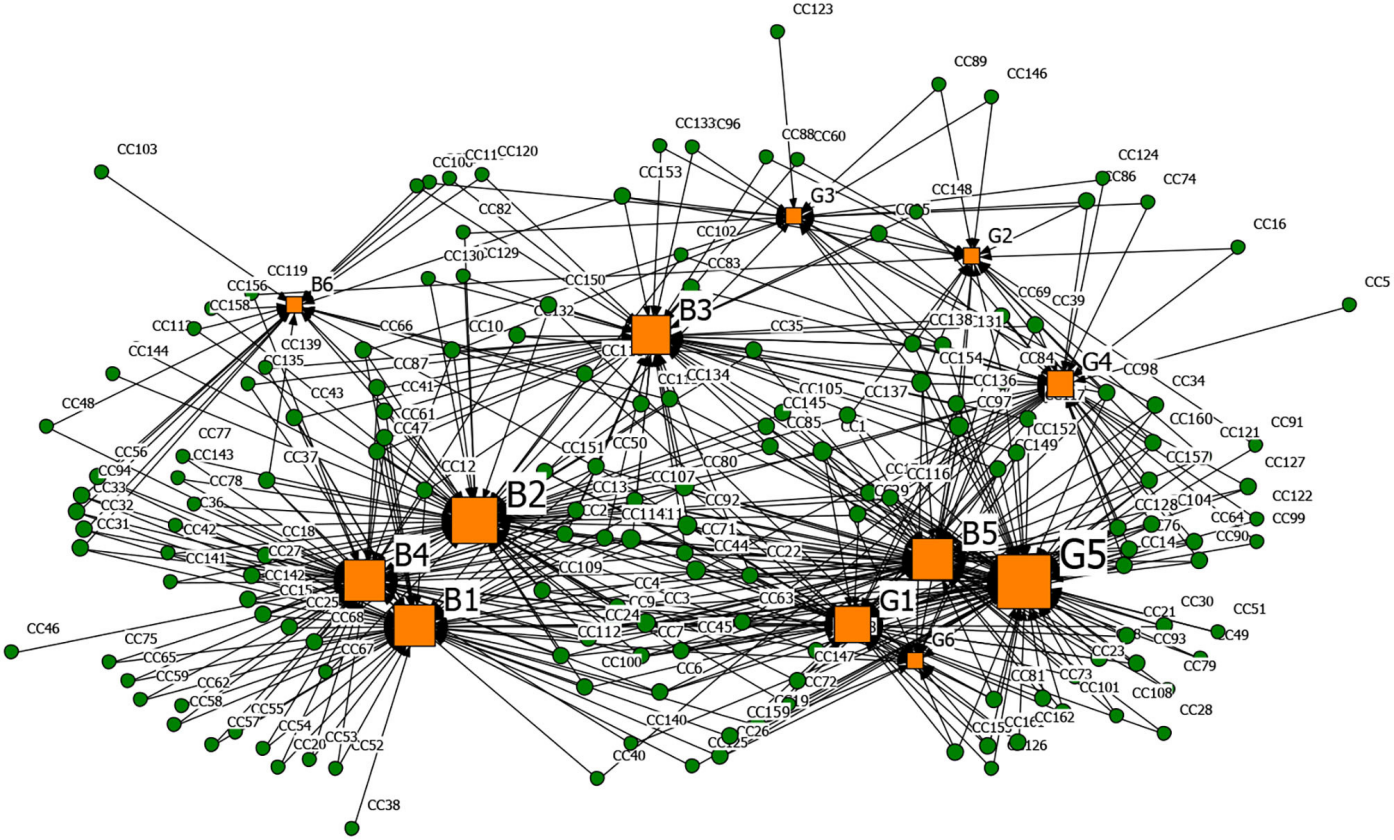
The graph of the CC-type rumor and generative factors network.

**Figure 7:**
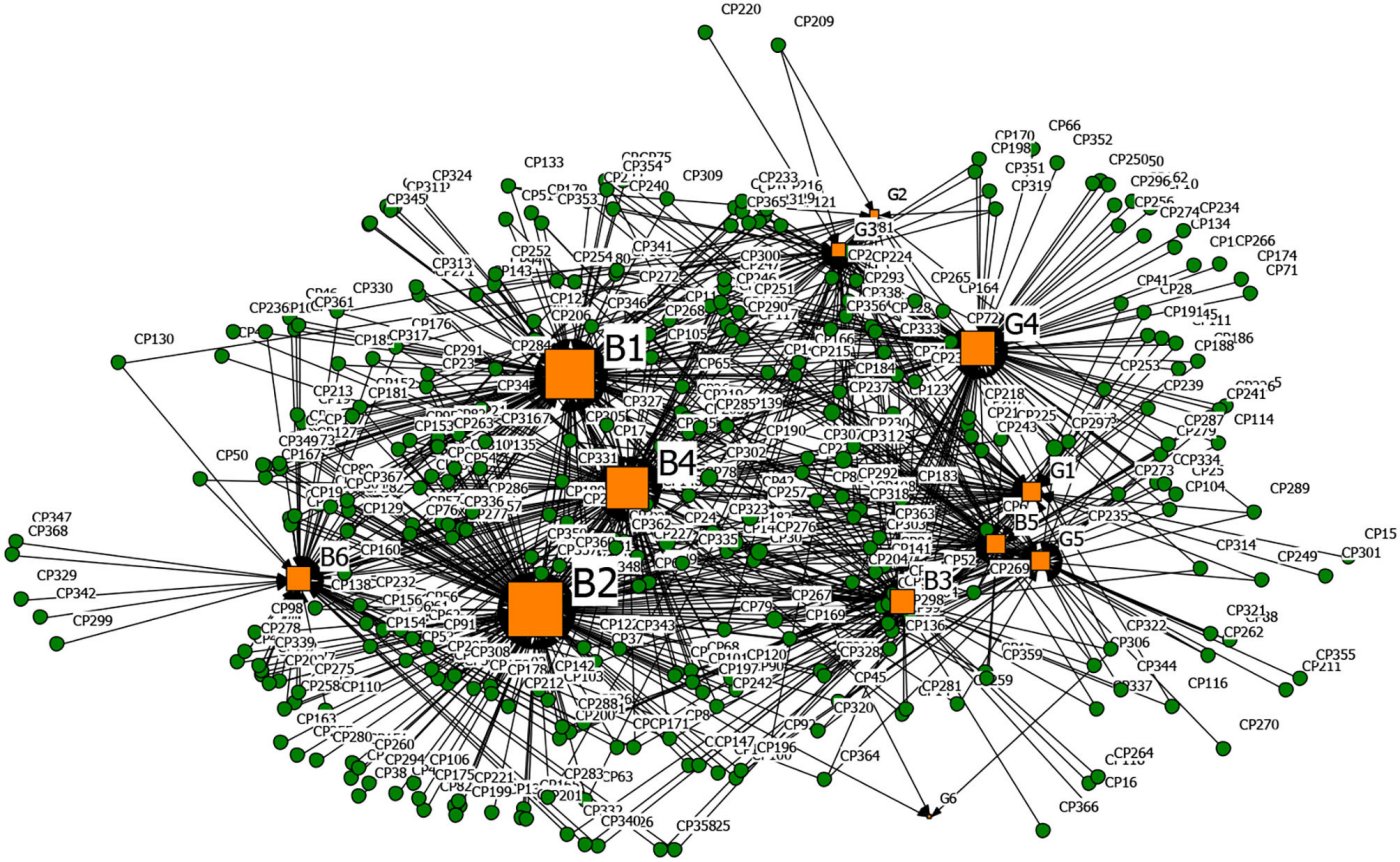
The graph of the CP-type rumor and generative factors network.

**Figure 8:**
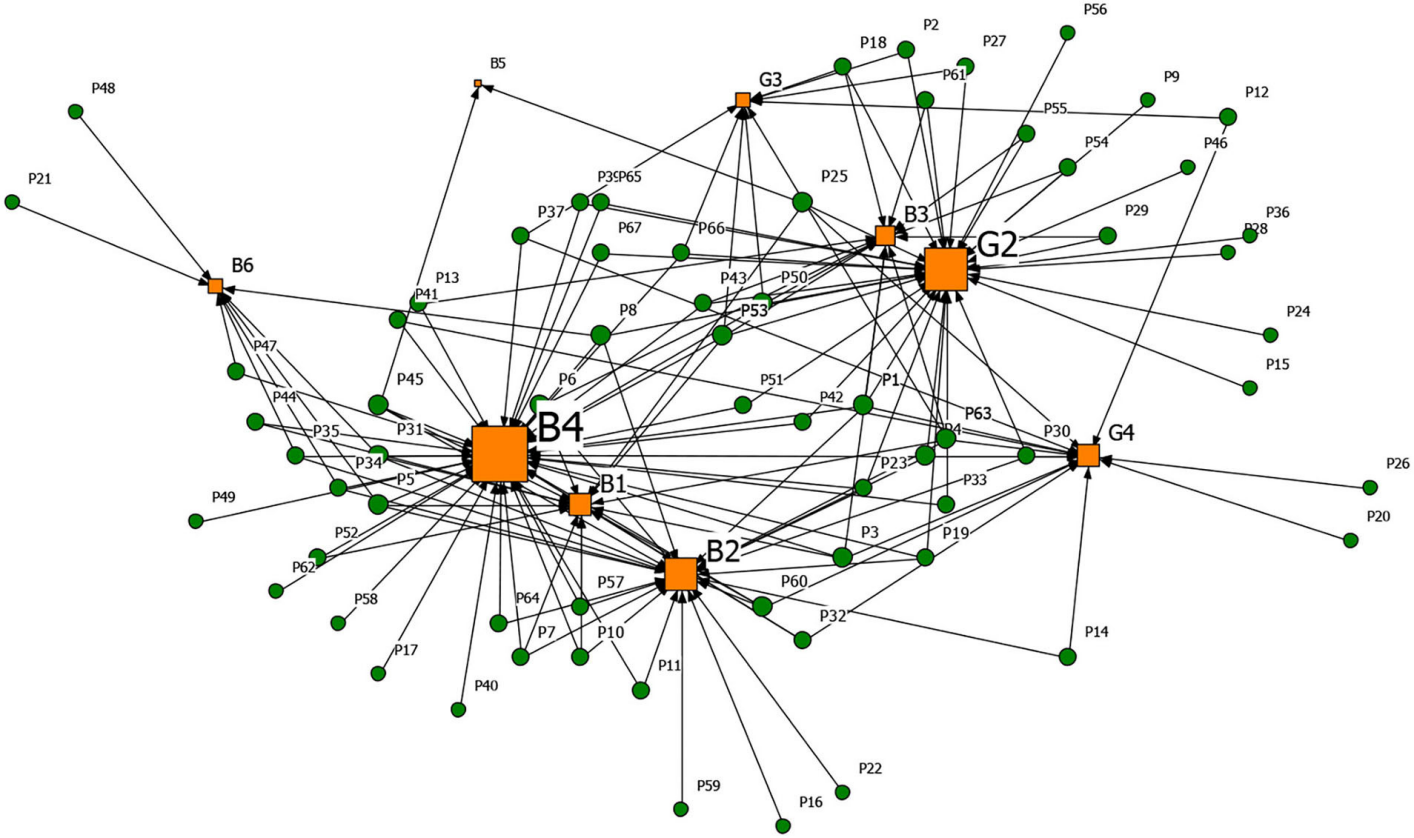
The graph of the P-type rumor and generative factors network.

**Figure 9:**
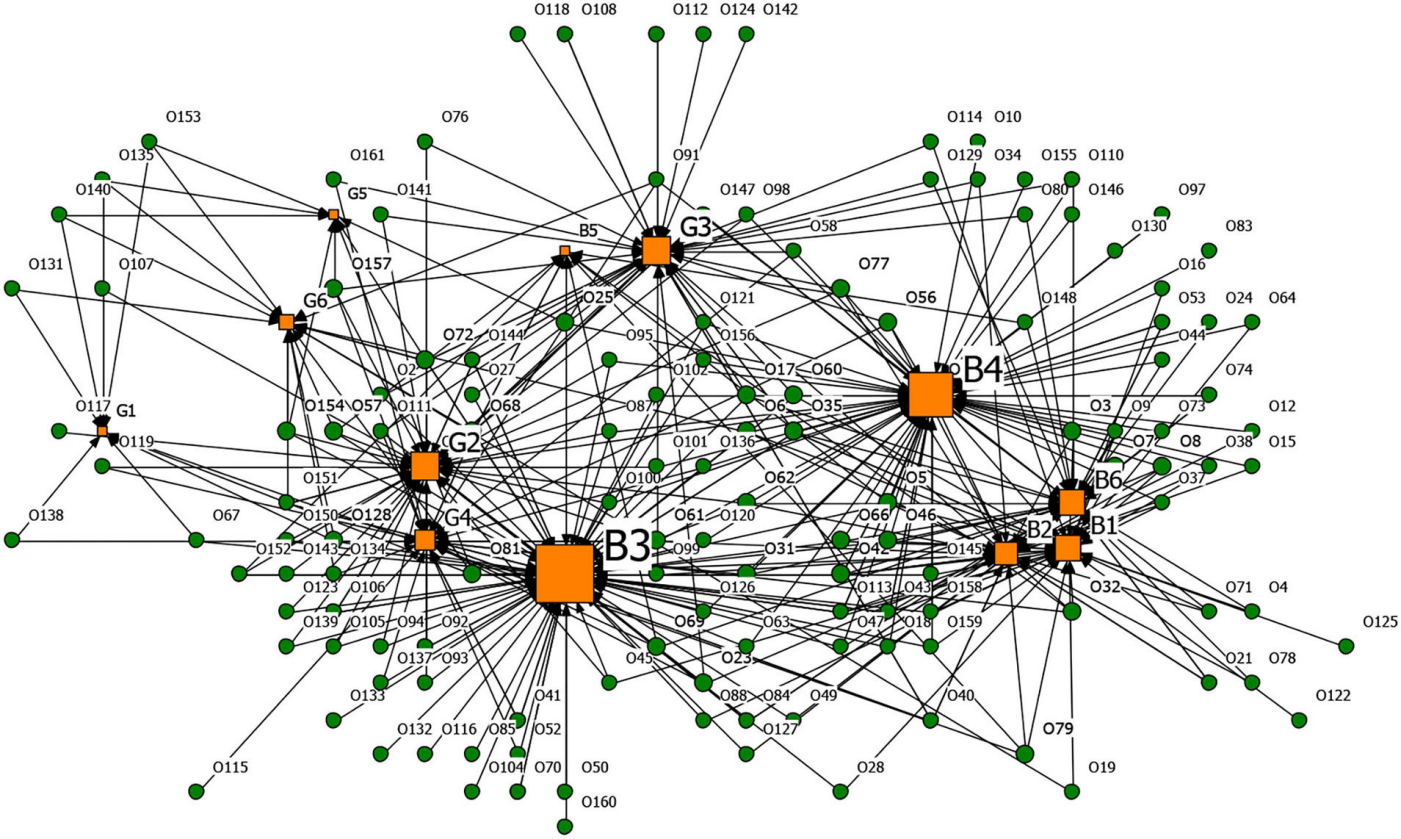
The graph of the O-type rumor and generative factors network.

**Figures 6**–**9** are the generating driver-rumor 2-mode network graphs corresponding to different rumor types, that is, CC-type rumor, CP-type rumor, P-type rumor, and O-type rumor.

From Figure 6, as for the CC-type rumors, node G5 (Deliberate sabotage by hostile forces) was the leading driver. The public spread the rumors that the coronavirus was a conspiracy without any factual evidence. Moreover, in ethnic minority areas, the rumor mongers fabricated that many ethnic minority compatriots were infected by the Han people and intended to stir up ethnic conflicts while creating a panic.

From Figure 7, as for the CP-type rumors, node G4 (To fool the public and seek social presence) also promoted the generation of rumors. Rumor makers fabricated rumors to gain public attention and satisfy their vanity. For example, some netizens lied to pick up many friends from Wuhan to come home and live in the community.

From Figure 8, as for the P-type rumors, the scale of node G2 (Grab economic interests) was second only to node B4 (Relatively lagging in information disclosure), which shows that the motivation of quite a several rumor makers was to ‘grab economic benefits.' With their interests in mind, some businesses created rumors to bid up the price of goods and make huge profits.

From Figure 9, as for the O-type rumors, node B6 (Well-intentioned reminder, arouse the public's attention to the epidemic) was that some rumor makers tried to raise others' awareness of epidemic prevention through rumor. Node B3 (Imperfect laws and regulations, inadequate network supervision) also contributed to the rumor making. The false epidemic prevention and control measures, such as false official epidemic prevention deployment documents and aircrafts spraying disinfectant, were still rampant after the official authorities repeatedly refuted them.

Table 12 presents the top five degree centrality of the drivers that can generate rumors in the 2-mode network. Combining Figures 6–9 and Table 12, it can be seen that node B4 (Relatively lagging in information disclosure) had a relatively large scale in the four types of rumors. According to the rumor circulation formula proposed by the famous American psychologist Allport and Postman (64), the importance of the content and the ambiguity in details are the keys to generating rumors. If the public does not get sufficient symmetry information in the face of public health emergencies, it will cause panic. In the early stage of the pandemic, the official information release channels were blocked, and the public was eager to learn about the situation, which prompted various rumors to emerge. Node B2 (Panic psychology) and node B1 (Uncertainty in the development of the epidemic) significantly impacted the CC-type rumors, CP-type rumors, and P-type rumors. After the outbreak of COVID-19, due to the lack of understanding of the coronavirus, Wuhan's severe situation after the lockdown of the city, and the shortage of medical resources and living materials, it was effortless to breed panic, making it easier for the public to believe the news that usually seemed unreal.

**Table 12:** Degree centrality of the top five driving factors that can generate rumors in the 2-mode network.

**Rumor type**	**Driving factors code**	**Degree centrality**
CC-type rumors	G5	76
	B2	64
	B4	58
	B5	58
	B1	56
CP-type rumors	B2	186
	B1	167
	B4	139
	G4	110
	B3	79
P-type rumors	B4	40
	G2	31
	B2	23
	B1	15
	G4	14
O-type rumors	B3	86
	B4	66
	G2	39
	G3	38
	B6	35

#### Spreading Driver-Rumor 2-Mode Network Analysis

**Figure 10:**
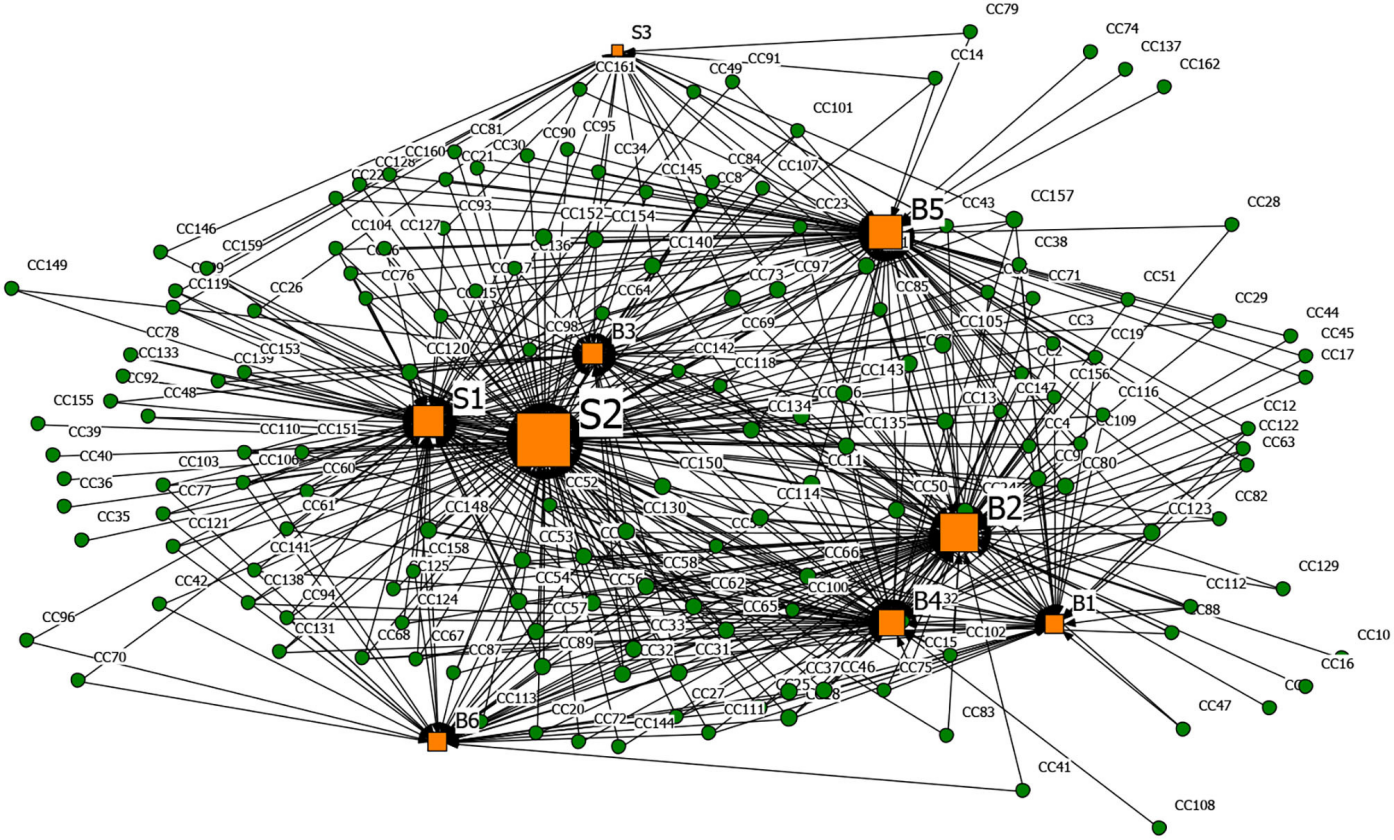
The graph of the CC-type rumor and spreading factors network.

**Figure 11:**
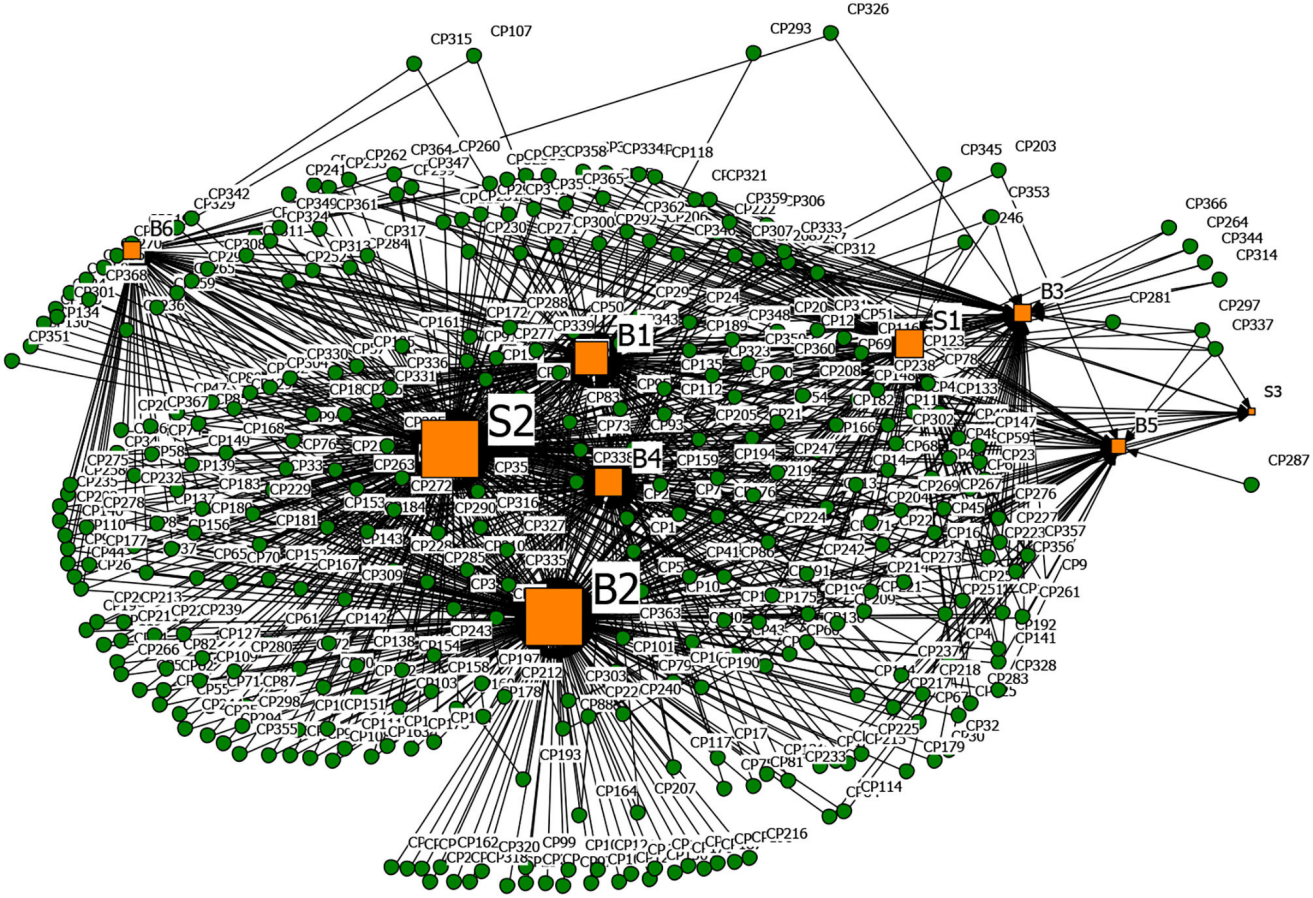
The graph of the CP-type rumor and spreading factors network.

**Figure 12:**
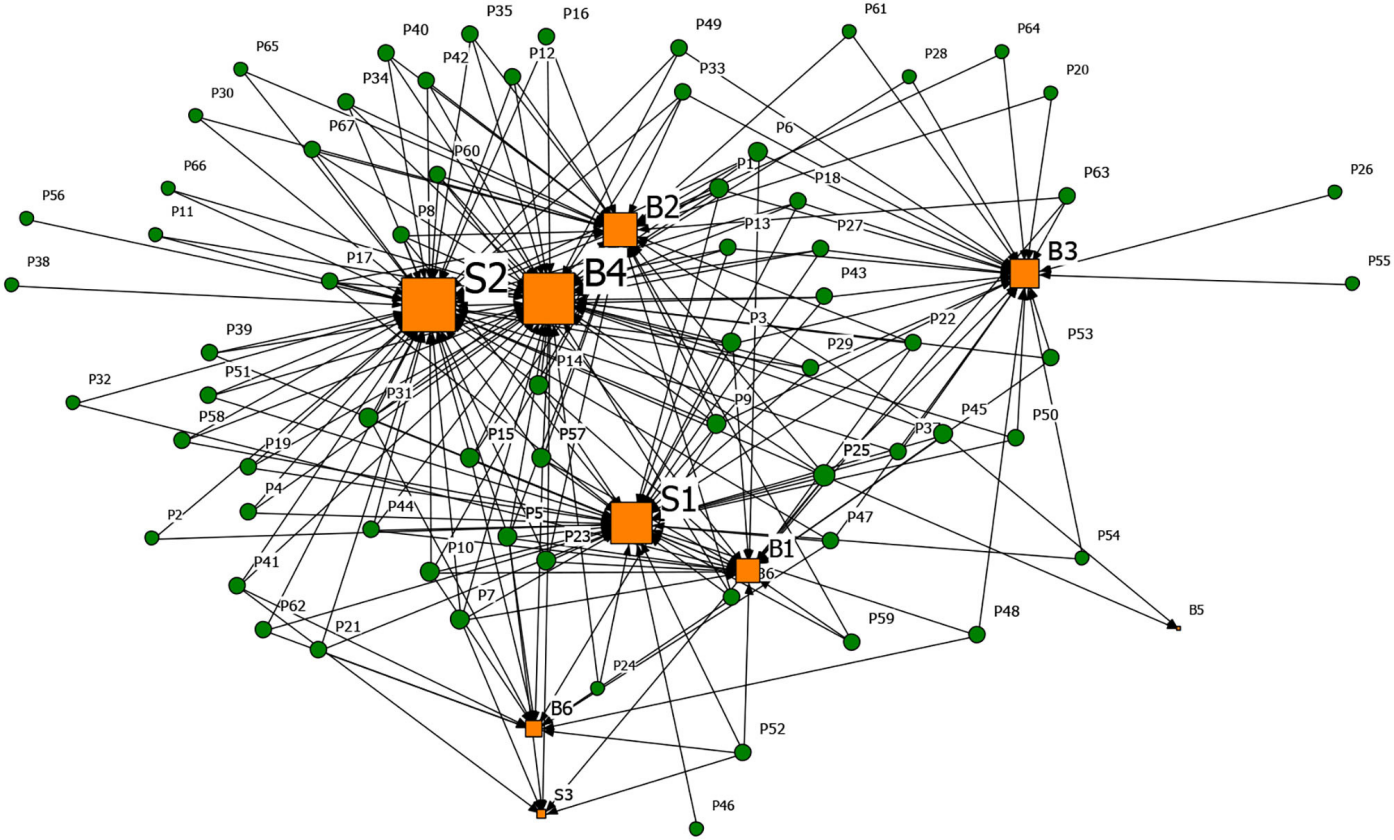
The graph of the P-type rumor and spreading factors network.

**Figure 13:**
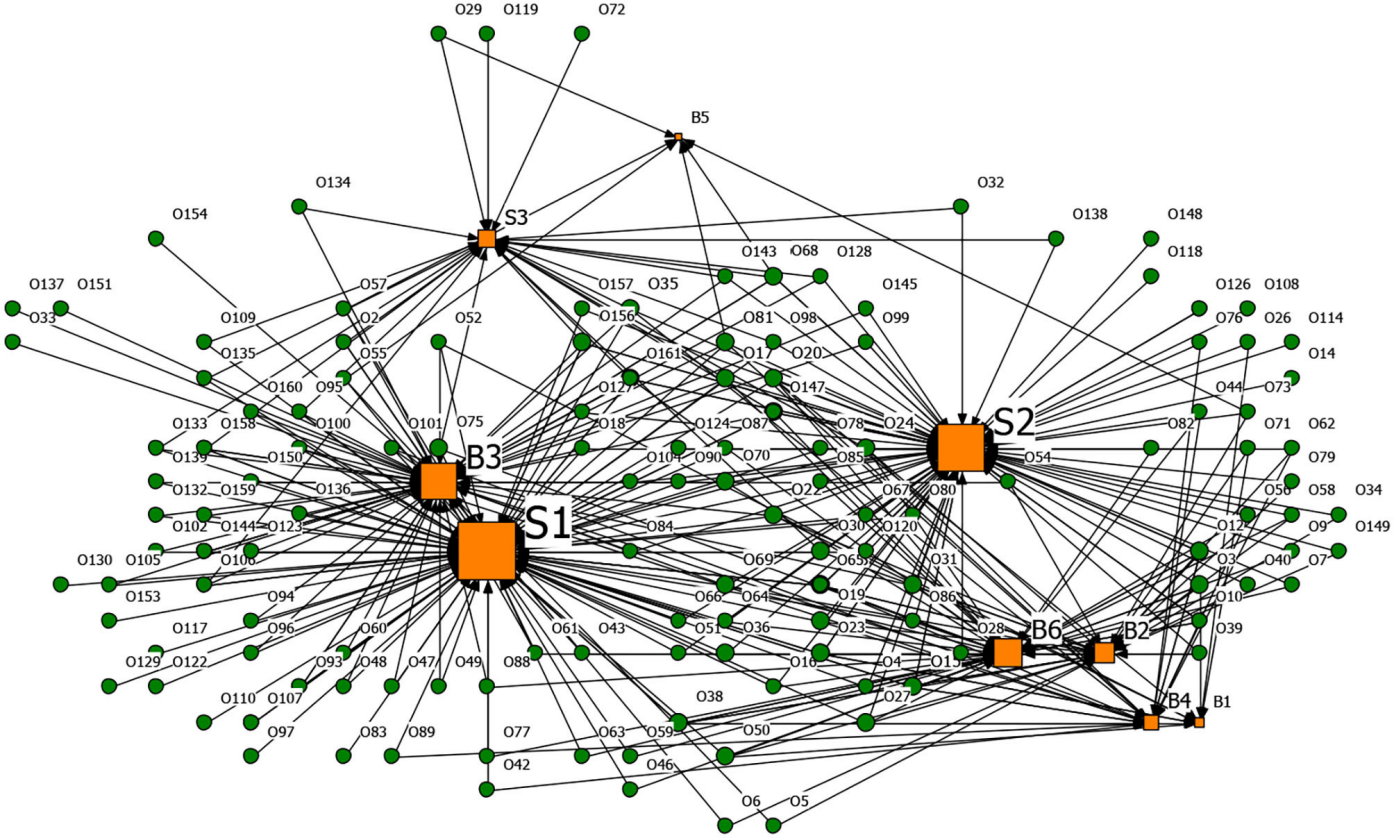
The graph of the O-type rumor and spreading factors network.

In the same way, the spreading driver-rumor 2-mode network results were obtained, as shown in Figures 10–13 and Table 13. It can be seen that nodes S2 (Low scientific literacy of the public, challenging to distinguish rumors), B2 (Panic psychology), and S1 (The confusing nature of rumors) were the main determinants for the dissemination of four types of rumors. In the early stage of the COVID-19 outbreak, the public knew little about coronavirus, the panic was most potent, and network information's screening ability declined. In node S1, the main form of P-type rumors was under the guise of authoritative experts, such as Academician Zhong Nanshan, where Creating Chaotic-type rumors and Other False-type rumors used ‘Urgent notice' and ‘Authoritative release' as the title, which made the public easily believe them and then spread them. Besides, node B4 (Relatively lagging in information disclosure) was another crucial driver for CC-type rumors, CP-type rumors, and P-type rumors. Looking back at the dynamic time axis of coronavirus development in some regions, some government departments' untimely release of information and the single release channel led to information asymmetry, which caused public understanding of information. As for the P-type rumors, the popularization of the new coronavirus-related knowledge was more important because ordinary people at home lack professional medical knowledge. Suppose that the authoritative professional institutions did not carry out extensive science popularization in time. In that case, the public would take the initiative to seek the correct information in line with personal cognitive ability and then believe, spread, and practice, thereby stimulating the spread of rumors. As for the CC-type rumors, node B5 (The government mishandled and broke the people's trust) shows that part of the public did not trust official information and was even deceived by conspiracy theories with ulterior motives. They resisted official information from the bottom of their hearts and believed that the official information must be false and trusted information from unknown sources in cyberspace.

**Table 13:** Degree centrality of the top five driving factors that can spread rumor in the 2-mode network.

**Rumor types**	**Driving factors code**	**Degree centrality**
CC-type rumors	S2	128
	B2	88
	B5	77
	S1	74
	B4	57
CP-type rumors	S2	284
	B2	281
	B1	153
	B4	129
	S1	122
P-type rumors	S2	49
	B4	46
	S1	38
	B2	31
	B3	25
O-type rumors	S1	102
	S2	84
	B3	64
	B6	44
	B2	32

## Strengths and Limitations

Studying and dealing with Internet rumors is a complex scientific issue, and this paper provides new insights into the existing literature. To the authors' best knowledge, this is the first study that attempts to explore the mechanisms of rumors in depth from various perspectives.

Although this study has provided exciting findings for theory and practice, some problems are still worth studying and solving. This paper takes Sina Weibo rumors during the COVID-19 outbreak as a sample, but the analysis of rumors needs to consider different emergency characteristics and social networking platforms. It is better to introduce rumors from different emergencies and social platforms for further analysis in future research.

## Conclusion

This is an in-depth and detailed study on rumors during the outbreak of novel coronavirus pneumonia. It can be concluded from the correlation analysis that the number of confirmed cases during the pandemic has a positive effect on the number of rumors. In detail, there was a change from a positive correlation to a negative correlation between the daily number of rumors and the cumulative confirmed cases, which indicated that the epidemic trend had generally become steady. The second significant finding was that the risk level of Creating Panic-type rumors was the highest and presented a sharp increase trend in the early stage of the epidemic. The last worth-highlighting point was the analysis of the rumor cause network, and we discovered that panic psychology and the lag of information disclosure were the crucial reasons for generating and propagating all kinds of rumors. In addition, the uncertainty of epidemic development played a vital role in the generation of rumors. The above-mentioned findings demonstrate that it is necessary to take timely and effective epidemic prevention and control measures and disclose information timely, which are conducive to reducing the public's negative mentality and controlling rumors. Besides, the confusing nature of rumors and the public's low scientific literacy were essential driving factors of rumor diffusion. The government needs to enhance the popularization of relevant science knowledge accordingly. The findings are conducive to authorities adopting targeted measures and further improving the level of social management.

## Data Availability Statement

The original contributions presented in the study are included in the article/supplementary material, further inquiries can be directed to the corresponding author/s.

## Author Contributions

WL: funding acquisition, validation, conceptualization, and writing—original draft. WZ and WL: formal analysis, methodology, and writing—review and editing. BG: writing—review and editing, funding acquisition, validation, and supervision. YH and HF: validation and writing—review and editing. All authors contributed to the article and approved the submitted version.

## Conflict of Interest

The authors declare that the research was conducted in the absence of any commercial or financial relationships that could be construed as a potential conflict of interest.

## Publisher's Note

All claims expressed in this article are solely those of the authors and do not necessarily represent those of their affiliated organizations, or those of the publisher, the editors and the reviewers. Any product that may be evaluated in this article, or claim that may be made by its manufacturer, is not guaranteed or endorsed by the publisher.

## Appendix The Survey Detail

**Part I Basic Information Survey**

**1. Your gender**

A. Male B. Female

**2. Your age**

A. Under 18 years old B. 18-30 years old C. 31-40 years old

D. 41-50 years old E. 51-60 years old F. Over 60 years old

**3. Your occupation type**

A. Civil servant or public institution worker B. Enterprise employee

C. Self-employed D. Students E. Others

**4. Your education level**

A. Junior high school and below B. Senior high school and junior college

C. Bachelor degree D. Graduate or above

**Part II Investigation on risk perception of Internet rumors related to COVID-19**

**5. Below are some representative rumors during the novel coronavirus pneumonia outbreak. Please judge the severity of the impact of these rumors on society according to your own honest opinion. 1 means no harm, 5 means the highest harm, and the degree of harm increases from 1 to 5**.

a) Yu Tian, the South China Seafood Market owner, whose father-in-law is vice-chairman of the Wuhan Huanan Seafood Market.

b) The medical team of Shanghai aid Hubei has no food to eat, only instant noodles.

c) Huang Yanling, a graduate of Wuhan Institute of Virology, Chinese Academy of Sciences, is the first “Patient Zero” infected with the new coronavirus.

d) Many other places are underreporting confirmed cases, possibly amounting to tens of thousands.

e) Zhaizhuang village, Xushui District, Baoding City, has three cases of infection, the village has been closed.

f) There are untreated corpses in a hospital in Wuhan.

g) The man is infected with COVID-19 after a 10-minute stop in Wuhan.

h) There are six people coming back from Korea in the qianshuiwan community of Tieling City.

i) Zhong Nanshan released that drinking more Dancong tea has a great effect on preventing pneumonia.

j) The N95 mask, which has been used for seven days, can continue to be used after being blown with a hairdryer or disinfected with alcohol.

k) Everyone needs to stay at home from 4:00 to 4:30 this afternoon. There will be planes spraying disinfectant.

l) A house was ignited by improper use of disinfectant alcohol in a community in Chengdu.
